# Central Venous Catheter for Treating Pseudomeningocele Compressing the Spinal Cord After Thoracic Ossification Surgery: Case Series

**DOI:** 10.1111/os.70254

**Published:** 2026-02-09

**Authors:** Chen Chen, Yue Xu, Yiqi Xu, Xiaojiang Sun, Yuan Nai, Jie Zhao, Jianmin Yuan, Changqing Zhao

**Affiliations:** ^1^ Shanghai Key Laboratory of Orthopaedic Implants, Department of Orthopaedic Surgery, Shanghai Ninth People's Hospital Shanghai Jiao Tong University School of Medicine Shanghai China; ^2^ Department of Orthopaedics, Changshu Hospital Nanjing University of Chinese Medicine Suzhou Jiangsu China; ^3^ Department of Orthopaedics Surgery, Fengcheng Hospital of Fengxian District Shanghai Ninth People's Hospital Shanghai China; ^4^ Department of Nursing, Shanghai Ninth People's Hospital Shanghai Jiao Tong University School of Medicine Shanghai China; ^5^ Department of Spinal Surgery, Second Hospital (Fuyang Fifth People's Hospital) Fuyang Normal University Fuyang Anhui China

**Keywords:** central venous catheter, ossifying disease, pseudomeningocele

## Abstract

**Background:**

Symptomatic pseudomeningocele (PMC) causing spinal cord compression is a severe complication following spinal surgery. Traditional management remains controversial, with surgical revision carrying significant risks. This study evaluates an innovative minimally invasive approach using central venous catheterization for percutaneous PMC drainage.

**Case Presentation:**

A multicenter case series included 17 patients with thoracic ossifying disease who developed PMC with neurological deterioration postoperatively. Under B‐ultrasound guidance, an experienced spinal surgeon performed percutaneous puncture and drainage of the PMC using a central venous catheter system. All patients achieved complete PMC drainage confirmed by MRI, with resolution of spinal cord compression.

**Conclusion:**

Ultrasound‐guided central venous catheter drainage is a safe, effective, and minimally invasive alternative for managing PMC‐induced spinal cord compression. This technique achieves rapid symptomatic relief, neurological recovery, and durable results without recurrence. Its successful extension to postoperative pseudocyst/abscess drainage suggests broad applicability in spinal complications.

## Graphical abstract

This study addresses a critical clinical challenge: the management of symptomatic pseudomeningocele (PMC) causing spinal cord compression following spinal surgery. Traditional treatments face limitations in efficacy and invasiveness. Here, we propose an innovative minimally invasive technique using central venous catheterization under ultrasound guidance for percutaneous drainage of PMC.

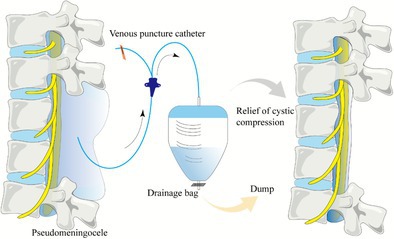

**Abbreviations**
CSF: cerebrospinal fluid
PMC: pseudomeningocele

## 1 Background

The concept of spinal pseudomeningocele (PMC) was initially documented in 1946, referring to a dural avulsion resulting in the primary leakage of cerebrospinal fluid (CSF) predominantly at the posterior aspect of the dural sac [1]. The classification of PMC includes congenital, iatrogenic, and traumatic origins. Trauma, bone fragments, or spinal surgical procedures (particularly laminectomy) can cause damage to the dura, resulting in the formation of PMC [2, 3]. If the arachnoid remains intact following dural damage, it will protrude through the dural defect, allowing CSF to accumulate within the arachnoid and form cysts. However, simultaneous tearing of the arachnoid and dura can result in CSF overflow into the epidural and paraspinal soft tissues. The absence of a dural covering enables partial absorption of CSF by these surrounding tissues. Nevertheless, as reactive fibrous tissue layers gradually develop, they impede CSF absorption, leading to accumulation in paraspinal soft tissues and subsequent compression of adjacent structures [2, 4]. The incidence of spinal cord compression caused by PMC is relatively low. However, in cases where the rupture functions as a unidirectional valve, there is a continuous rise in pressure within the pseudocyst, resulting in compression of the spinal cord or nerve roots. As a result, patients primarily present with limb pain, numbness, or functional impairment, along with headache associated with reduced CSF and intracranial pressure [2]. In summary, it is imperative for spine surgeons to promptly diagnose and administer appropriate treatment for PMC.

The current consensus is that conservative management can be attempted for the majority of asymptomatic PMC cases, while the optimal treatment approach for symptomatic PMC remains a subject of controversy [5, 6, 7]. Although certain surgeons advocate surgical intervention for all patients with symptomatic PMC, like CSF diversion drainage, shunting and epidural blood patch [8, 9], others recommend a relative conservative treatment approach [10, 11]. The catheter drainage procedure is a minimally invasive and efficacious interventional therapy, which is more and more compelling, primarily employed in patients presenting with prominent symptoms, inadequate response to conservative treatment, or complications [5]. In this study, successful management of postoperative PMC was achieved through central venous catheterization and drainage in 17 patients who had undergone spinal surgery for ossifying disease from multicenter. This intervention led to significant improvement in their clinical symptoms and ensured long‐term absence of recurrence. The purpose of our study is to conduct a comprehensive analysis of the case series and present advanced central venous catheter techniques.

## 2 Methods

This present study observed 17 patients who underwent surgical treatment for thoracic ligament ossification between 2016 and 2024, subsequently exhibiting the development of localized epidural cysts that exerted compression on the spinal cord, thereby exacerbating neurological symptoms. These patients ranged in age from 40 to 77 years, including nine male patients and eight female patients. For the patients included in the observation, we observed the postoperative neurological symptoms, the time of symptom occurrence and the corresponding neurological symptoms, reviewed the MRI, evaluated the puncture drainage time, symptom improvement, and posttreatment MRI.

**FIGURE 1:**
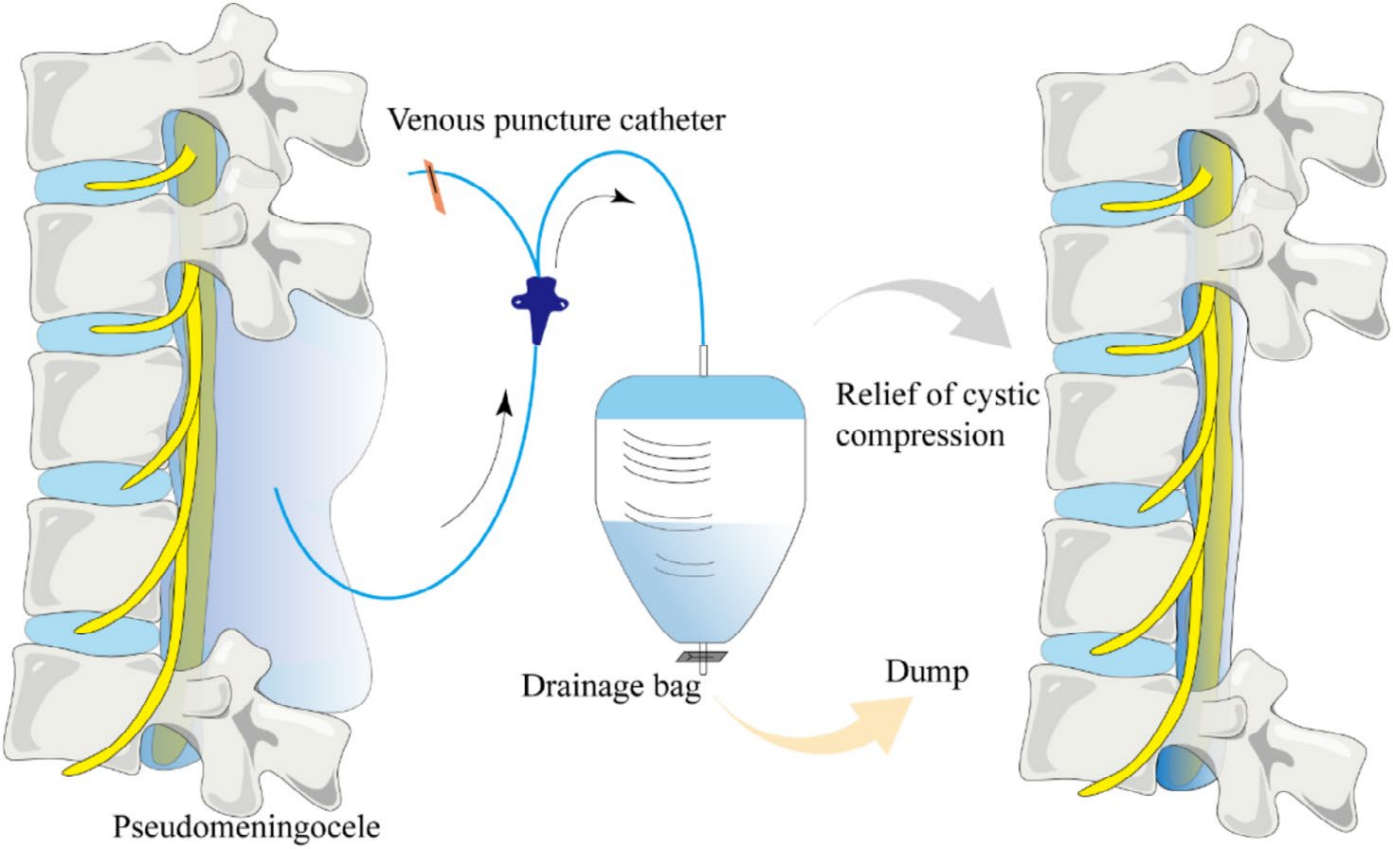
The illustration shows deep venous puncture catheterization for PMC and successful relief of cystic compression.

Following the diagnosis of PMC, a central vein puncture catheter was used by an experienced spinal surgeon under B‐ultrasound guidance to puncture and drain the cyst (Figure 1). The standard protocol of treatment is as follows:

Firstly, the hands of the surgeon were washed, sterilized, and a surgical drape was laid in accordance with aseptic principles. Subsequently, under B‐ultrasound guidance, the puncture needle was positioned at the cyst site while placing a guide wire accordingly. The skin was then expanded before inserting an intravenous catheter into the cyst site along the guide wire, with the catheter tip at the center of the PMC. Finally, the catheter was secured and disinfected after a common drainage bag was connected to the end of the catheter. The inserted catheter is a single‐lumen catheter, model 18G, with an inner diameter of approximately 0.9 mm and an outer diameter of approximately 1.27 mm. The indwelling duration of the drainage tube is typically approximately 10 days; however, the precise timing for removal should be determined based on a comprehensive assessment of soft tissue healing, preferably through magnetic resonance imaging. Prolonged catheterization may increase the risk of retrograde infection; therefore, a careful evaluation of the potential benefits and risks is essential. Upon successful placement of the central venous catheter, aspiration drainage can be initiated immediately, with close monitoring of any changes in the patient's neurological status. The daily drainage volume should be adjusted according to the patient's clinical tolerance, aiming to prevent symptoms associated with low intracranial pressure. As a general guideline, the daily drainage volume should not exceed 200 mL. Patients were encouraged to ambulate actively during the drainage period. Following the MRI imaging examination, which confirmed the absence of the PMC, the catheter was maintained in situ for an additional 2 to 3 days prior to removal.

## 3 Case Presentation

The average age of these patients is 63 years, the average time of symptom onset and drainage placement is 27.6 and 13.8 days. The detailed illustration will be centered on Patient1 and Patient2.

### 3.1 Patient1

The patient, a 56‐year‐old female with a medical history of hypertension, sleep apnea hypopnea syndrome, and tuberculosis, presented to our hospital in 2022 due to a 1‐year history of walking instability and a 7‐month sensation of tightness in the chest and abdomen. Diagnostic findings revealed ossification of the posterior longitudinal ligament and spinal stenosis in the thoracic vertebrae. T1–T7 total laminectomy along with left T1/T2 pedicle screw fixation was performed in our hospital. Following surgery, there was significant improvement in symptoms leading to subsequent discharge. However, 6 months postsurgery, the patient experienced pain and numbness in both lower limbs without any apparent cause. The left lower limb exhibited pain radiating from the buttocks to the back of the thigh, lateral leg, back, and sole of the foot bilaterally. Left limb mobility was restricted with weak hip flexion, knee weakness, and left foot drop. Muscle strength examination of the left lower limb: iliopsoas (Grade 2), quadriceps (Grade 2), anterior/posterior tibial muscles (Grade 0), toe dorsal extensor muscle (Grade 0), plantar flexor muscle (Grade 3). Other muscle strength is normal. Decreased sensation below xiphoid plane on the left side was noted along with a positive bilateral Babinski sign indicating abnormal reflexes; decreased bilateral knee jerk reflexes as well as ankle reflexes; anal sphincter contraction remained intact.

### 3.2 Patient2

The patient is a 40‐year‐old male with a medical history of undergoing surgery for ossification of the thoracic posterior longitudinal ligament and was admitted this time due to trauma. The patient exhibits bilateral weakness in the lower extremities, decreased sensation below the level of the costal arch, and an inability to ambulate. Muscle strength assessment reveals Grade 3 quadriceps muscle strength in both lower extremities and Grade 3 anterior and posterior tibial muscle strength. There is increased muscle tone observed in both lower limbs along with a positive bilateral Babinski sign. Anal sphincter contractile function remains intact.

**FIGURE 2:**
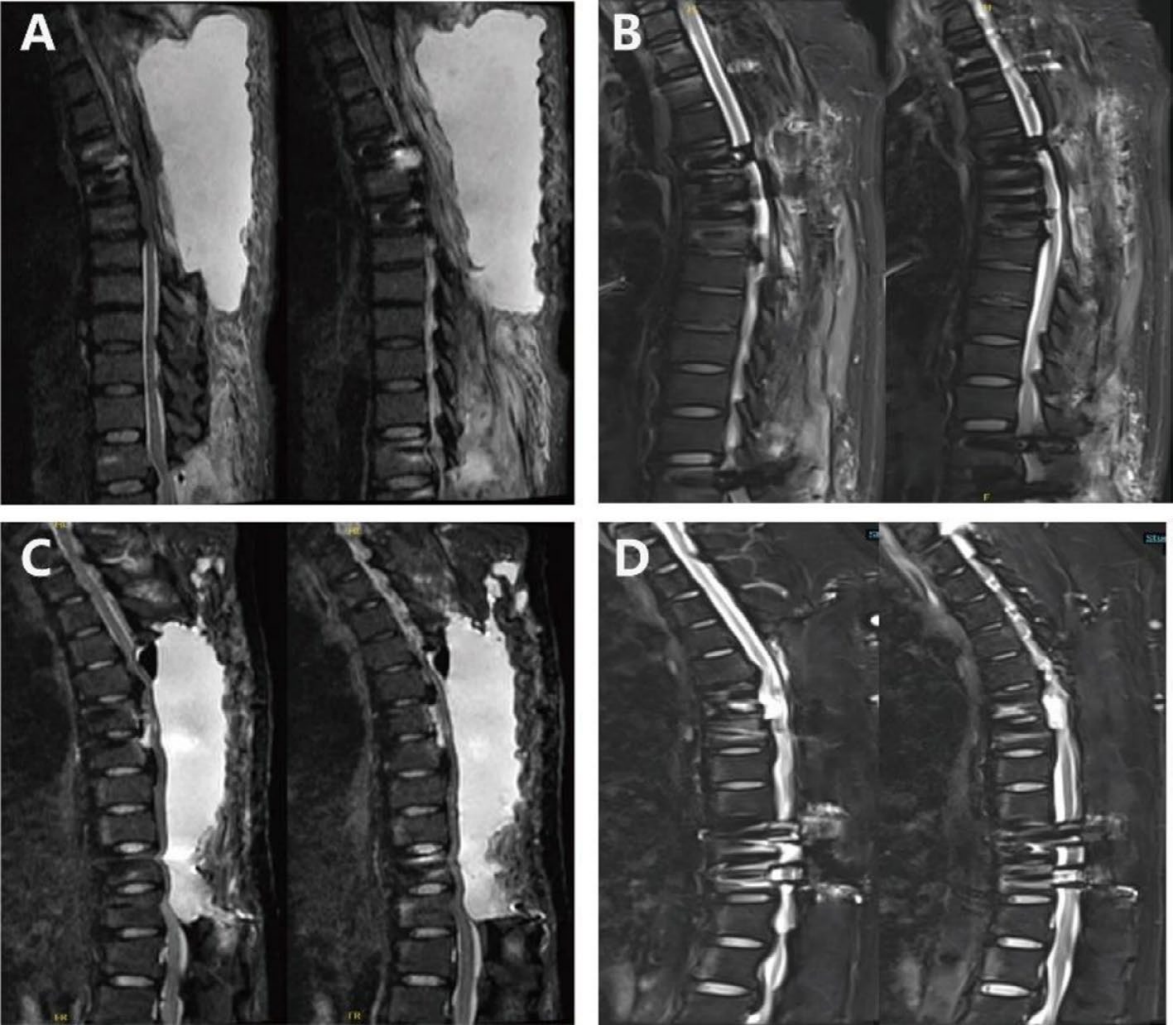
The MRI image (A) depicts the prepuncture condition of the patient in case1, wherein the high‐density region appearing as a bright white area represents PMC. Subsequently, (B) demonstrates a significant disappearance of the cyst after puncture. (C and D) show the patient in case2 before and after the puncture.

Both of the aforementioned patients underwent intravenous catheter puncture and drainage therapy guided by B‐ultrasound upon admission. The MRI images before and after the puncture are illustrated in Figure 2. The sagittal MRI images depicted in Figure 2A,C vividly illustrate a well‐defined irregular‐shaped hyperintense area present in both patients before puncture, closely resembling CSF signal intensity within the dural sac. This finding strongly indicates substantial CSF accumulation at this location, aligning with PMC's underlying pathogenesis. Dural sac disruption causes CSF efflux into extra‐spinal canal paravertebral space and subcutaneous tissue, leading to compression on paravertebral tissues. Encouragingly, the PMC disappeared and the paravertebral tissue at the primary site returned to a relatively normal anatomical position after central venous catheterization and drainage in these two patients, as demonstrated in Figure 2B,D. Both patients experienced significant symptom relief, and no recurrence of PMC has been observed thus far.

The remaining 15 patients have shown significant improvement after receiving the same treatment and have remained recurrence‐free during the 2‐year follow‐up period. Among the 17 patients included in our clinical study, there were no cases of catheter blockage or displacement. The clinical information of all patients, including gender, age, postoperative ASIA grading, as well as pre‐ and postpuncture ASIA grading, has been summarized in Table 1.

**TABLE 1:** The basic and clinical information of patients enrolled.

No.	Gender	Age	Operative segment	Postoperative ASIA grading	Time of postoperative neurological symptoms (day)	ASIA grading	Days of drainage (day)	ASIA grading after drainage
001	Female	56	T1–T7	E	28	C	11	E
002	Male	40	T4–T10	D	43	C	17	D
003	Male	67	T11–L1	E	16	C	10	E
004	Male	72	T2–T6	E	28	B	21	D
005	Female	68	T10–T12	E	20	D	14	E
006	Female	73	T3–T4&T8–T10	D	24	C	12	D
007	Female	77	T2–T6	C	38	B	18	C
008	Male	54	T9–T11	E	42	D	14	E
009	Male	63	T5–T7	E	20	D	15	E
010	Female	72	T6–T9	D	18	D	10	D
011	Male	65	T8–T9	E	23	C	16	D
012	Female	70	T6–T8	E	39	D	12	E
013	Female	58	T1–T4	D	27	C	15	D
014	Male	69	T8–T10	D	25	D	10	D
015	Male	57	T4–T6	E	36	D	14	E
016	Male	49	T9–L1	E	19	D	13	E
017	Female	66	T4–T5&T9–T10	D	24	D	12	D

## 4 Discussion and Conclusions

This study aims to explore an innovative technique, applying central venous catheterization for the treatment of large pseudocysts formed after CSF leaks following spinal surgery. Postoperative CSF leaks after spinal surgery are a serious complication, and the most severe long‐term complication is the formation of pseudocysts that cause neural compression symptoms, increasing patient suffering and healthcare costs [12, 13, 14]. Therefore, finding a new and effective treatment method is crucial for improving patient outcomes.

Our research results indicate that central venous catheter technology has shown significant efficacy in treating pseudocysts resulting from post‐CSF leak. Compared to traditional open surgery, this technique has the advantages of being minimally invasive, having a faster recovery, and fewer complications. These findings align with recent research trends, which emphasize improving treatment outcomes while reducing invasiveness [15, 16, 17, 18]. The main advantage lies in its minimally invasive nature, which not only reduces physical trauma to the patient but also shortens hospital stays. Moreover, the application of this technology reduces surgery‐related complications such as infection and bleeding, which is particularly important for high‐risk patients. Compared to traditional treatments where most doctors would opt for puncture drainage followed by compression bandaging and surgical treatment, both have significant limitations; our study highlights the potential of minimally invasive technology in this field [19, 20].

However, the mechanisms underlying the success of central venous catheter drainage are more complex than simple fluid removal. (1) Valve‐effect interruption: The formation of PMC is primarily attributed to the synergistic effect of the “one‐way valve mechanism” and progressive cerebrospinal fluid accumulation. Central venous catheter drainage effectively reduces intracapsular pressure and disrupts the one‐way valve phenomenon, thereby establishing a bidirectional pressure equilibrium at the dural defect site. This facilitates optimal conditions for the development and eventual closure of fibrous scar tissue [21]. (2) Biomaterial advantage: The central venous catheter is fabricated from polyurethane material exhibiting a surface negative charge [22, 23]. It demonstrates a lower protein adsorption rate compared to silicone catheters, thereby significantly reducing the risk of distal catheter occlusion caused by protein deposition. This property provides the essential material foundation for achieving a zero catheter occlusion rate in this study. Additionally, we have conducted this procedure in patients who experienced nerve compression caused by acute epidural pseudocyst following lumbar surgery, as well as nerve compression resulting from infection‐related abscess after spinal surgery. An elderly woman undergoing lumbar surgery developed epidural pseudocyst compression as a result of postoperative anticoagulant treatment, leading to exacerbated neurological symptoms in her lower extremities. We utilized B‐ultrasound guidance for precise localization of the pseudocyst and subsequently inserted a deep venous catheter following negative pressure aspiration to ameliorate the patient's neurological symptoms. Another patient was an elderly male undergoing lumbar spine surgery. Following the development of a postoperative abscess caused by infection and nerve compression, the abscess was accurately located using B‐ultrasound guidance. A deep venous catheter was then inserted into the center of the abscess under precise placement, followed by application of negative pressure suction in combination with administration of sensitive antibiotics. After treatment, the abscess volume of the patient was reduced, inflammation indexes were changed, and neurological symptoms were improved.

Although our research results are encouraging, we are also aware of the limitations of the technique. First, we have only conducted case reports, which may limit the generalizability of the results. Second, central venous catheter technology requires highly specialized ultrasound techniques, which may limit its application in resource‐limited settings. Future research needs to validate these results in a larger patient population and explore how to overcome these challenges. At the same time, our exploratory research found that the technology has certain feasibility for the drainage of abscesses after spinal surgery infection and for the drainage of pseudocyst after spinal surgery.

## 5 Conclusion

In summary, central venous catheter technology has shown significant potential in treating PMC caused by CSF leaks after spinal surgery. Despite some limitations, the development of this technique offers patients a safer and more effective treatment option. Based on our research results, we recommend that clinicians consider central venous catheter technology as an alternative treatment method for PMC formed after CSF leaks following spinal surgery.

## Author Contributions

**Chen Chen**, **Jianmin Yuan**, and **Changqing Zhao:** study conception and design. **Yue Xu** and **Jie Zhao:** data collection. **Yiqi Xu**, **Xiaojiang Sun**, **Yuan Nai:** analysis and interpretation of results. **Yiqi Xu** and **Chen Chen:** draft manuscript preparation. All authors reviewed the results and approved the final version of the manuscript.

## Funding

This study was supported by the Shanghai Jiao Tong University Medical interdisciplinary research Foundation (YG2023QNA22), the Shanghai Municipal Health Commission (202040329), National Natural Science Foundation of China (82502945), Natural Science Foundation of Shanghai (24ZR1442300) and Shanghai Leading Talent Program of Eastern Talent Plan (QNWS2024007).

## Conflicts of Interest

The authors declare no conflicts of interest.

## Data Availability

Data sharing is not applicable to this article as no datasets were generated or analysed during the current study.
